# Give me a SINE: how Selective Inhibitors of Nuclear Export modulate autophagy and aging

**DOI:** 10.1080/23723556.2018.1502511

**Published:** 2018-08-17

**Authors:** A.V. Kumar, T.G. Thakurta, M.J. Silvestrini, J.R. Johnson, R.A. Reenan, L.R. Lapierre

**Affiliations:** Department of Molecular Biology, Cell Biology and Biochemistry, Brown University, Providence, RI, USA

**Keywords:** Autophagy, CRM1, XPO1, TFEB, Nuclear export

## Abstract

Autophagy is a cellular recycling process leading to lysosomal degradation of damaged macromolecules, which can protect cells against aging. The transcription factor EB (TFEB), a major transcriptional regulator of genes involved in autophagy and lysosomal function, is emerging as an attractive target for pharmacological modulation. Recently, we demonstrated that inhibiting the function of nuclear export protein exportin 1 (XPO1 or CRM1) with RNAi or with selective inhibitors of nuclear export (SINE) results in the nuclear enrichment of TFEB and enhancement of autophagy in model organisms and human cells. In addition to current efforts to validate the use of SINE in cancer therapies, our work highlights the potential benefits of these drugs toward improving outcomes in neurodegenerative diseases and aging.

## Commentary

The partitioning of proteins between the nucleus and cytoplasm is modulated by the nuclear pore and karyopherins that carry specific proteins in and out of the nucleus. This dynamic process is driven by the interaction between karyopherins and cargoes, which are powered by ras-related nuclear protein-GTPases (Ran-GTPases) for transport across the nuclear pore (Reviewed in^1^). Importins mediate the import of proteins with nuclear localization sequences while exportins facilitate the nuclear export of proteins containing nuclear export sequences. By altering the function of importins and/or exportins, the nuclear protein landscape can be modulated and transcriptional and functional outputs can be modified.

We became interested in the role of karyopherins while investigating the regulation of the nuclear localization of the autophagy-regulating transcription factor EB (TFEB). Our laboratory has been investigating the nematode *C. elegans* ortholog of TFEB, called HLH-30, which modulates autophagy, lysosomal biogenesis and lifespan in a variety of long-lived mutants^2^. Indeed, one of the major features of long-lived animals is the enrichment of specific transcription factors in the nucleus. To find new genetic modifiers of HLH-30/TFEB, we screened for genes that, when silenced with RNAi, would lead to the enrichment of HLH-30 in the nucleus. The top candidate from our unbiased genome-wide RNAi screen was the nuclear export protein XPO-1 (XPO1, CRM1 or exportin 1 in mammals). Silencing *xpo-1* led to nuclear enrichment of HLH-30 in all RNAi-treated animals^3^. Further characterization in nematodes led to the discovery that XPO-1 modulates HLH-30 function, autophagy and lifespan. Specifically, key transcriptional targets of HLH-30/TFEB were enhanced, which conferred increases in autophagosome and autolysosome formation. Autophagy induction by reducing XPO-1 was accompanied by an increase in the autophagy protein LGG-1/microtubule-associated protein 1A/1B-light chain 3 (LC3), as observed by other groups^4,5^. In addition, our findings provide functional support to proteomic analyses establishing TFEB as a potential cargo for XPO1-mediated nuclear export^6^.

As autophagy induction is a longevity-associated process^7^, we investigated the effect of reducing XPO-1 on aging and found that it extended lifespan significantly in nematodes and in a manner similar to established longevity models. Indeed, we found that long-lived animals have reduced expression of *xpo-1*, suggesting that reduced nuclear export may improve the ability of these animals to maintain a longevity-associated nuclear protein profile. We anticipated that inhibiting nuclear export pharmacologically could possibly mimic the effect seen using *xpo-1* RNAi. In addition, there has been increased interest in finding autophagy modulators that bypass the need to interfere with the mechanistic target of rapamycin (mTOR) pathway in order to avoid unwanted side effects^8^. Therefore, by inhibiting XPO1 activity and enriching the nucleus with TFEB, we reasoned that we might avoid affecting mTOR altogether.

The expression of XPO1 is increased in a variety of cancer types and the consequent enhanced nuclear export leads to cytoplasmic re-partitioning of numerous cargoes, including many tumor suppressing proteins such as p21, p27, p53, p73, breast cancer gene (BRCA 1/2), retinoblastoma protein (pRB), protein phosphatase 2A (PP2A) and forkhead box proteins (FOXO) (Reviewed in^9^). To counteract the increase in XPO1 activity, several selective inhibitors of nuclear export (SINE) have been developed and, combined with other chemotherapeutic agents, these compounds have shown promising effects against various cancers (Reviewed in^10^). In our study, we found that SINE compounds, and in particular selinexor (KPT-330, Karyopharm Therapeutics) had beneficial effects on the lifespan of nematodes and ALS-afflicted flies, recapitulating our experiments with *xpo-1* RNAi. In HeLa cells, SINE compounds enhanced autophagy and lysosomal biogenesis in a TFEB-dependent manner demonstrating a conserved mechanism by which HLH-30/TFEB activity is modulated by nuclear export. In contrast to the mTOR inhibitor Torin1, SINE did not reduce mTOR activity, indicating that XPO1 inhibition is a viable avenue for stimulating autophagy without affecting mTOR. Although mTOR activity seems intact upon SINE treatment, mTOR is also likely to accumulate in the nucleus as it is an XPO1 cargo^6^. As observed in the cytosol, it is possible that nuclear mTOR phosphorylates TFEB and affects its nuclear export, which is prevented due to XPO1 inhibition by SINE compounds. Considering the relatively safe profile of SINE, it will be interesting to learn how efficient these compounds may be at enhancing autophagy and lysosomal function in mice and humans.

**Figure 1.**
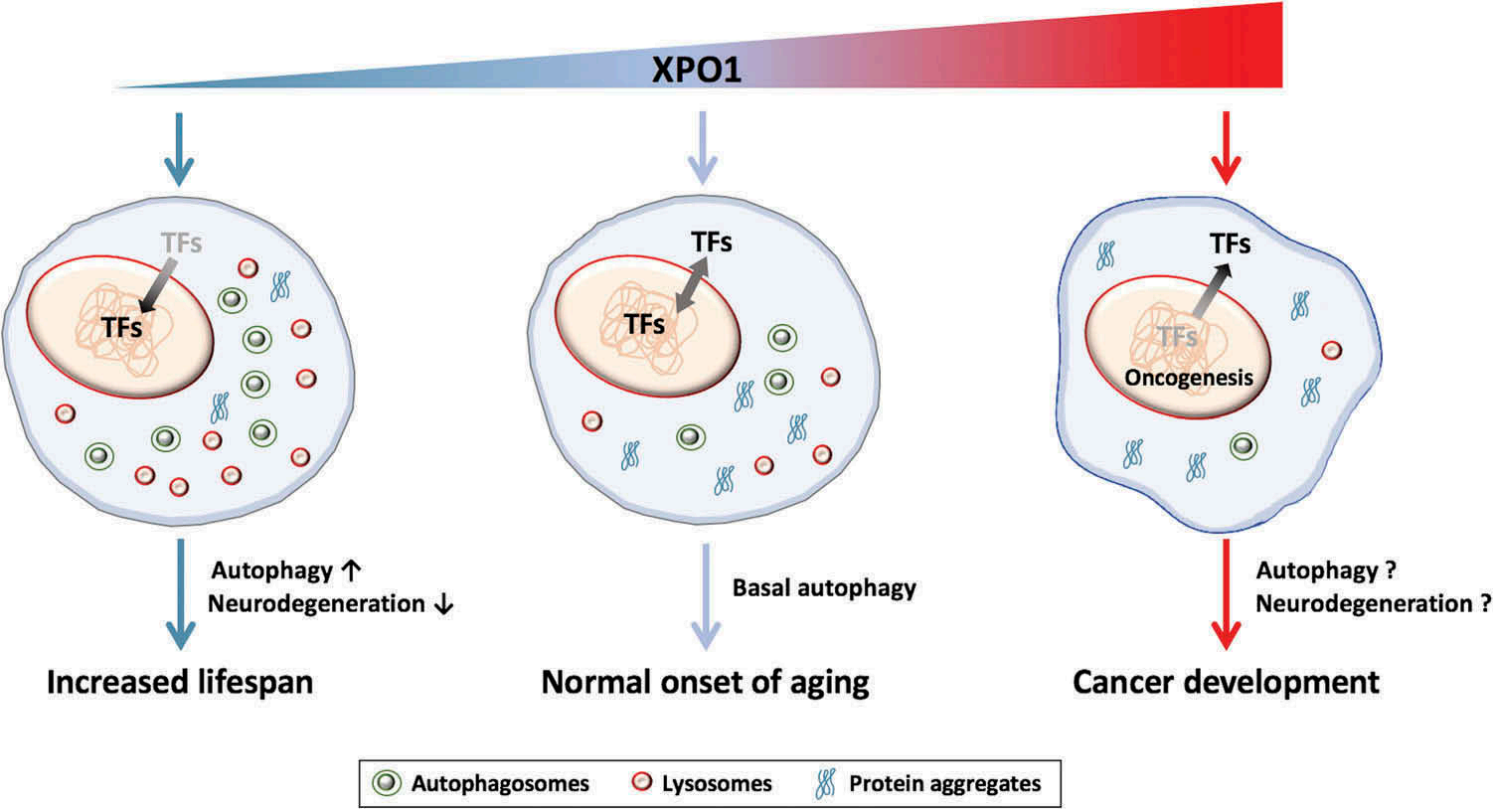
**XPO1 levels modulate autophagy and aging**. Exportin 1 (XPO1) acts a pivot that governs cell survival on one hand and cancer progression on the other. Under unperturbed conditions, XPO1-mediated nuclear export of transcription factors (TFs) maintains basal autophagy, which leads to normal onset of aging. When XPO1 is upregulated, as seen in some cancers, there is excessive loss of TFs including tumor suppressor factors from the nucleus. This aberrant level of nuclear export promotes oncogenic genes and cancer progression. Conversely, XPO1 inhibition causes enrichment of TFs in the nucleus that promote autophagy and clearance of protein aggregates leading to increased longevity.

Overall, our study highlights the role of XPO1 in autophagy and longevity modulation. In a cellular context (Figure 1), we propose that XPO1 levels represent a pivot between survival and growth by affecting sets of transcription factors (TFs), including TFEB and FOXO, which in turn improve autophagic and lysosomal function. Re-partitioning of other proteins in the nucleus may also contribute to improving longevity. When XPO1 levels increase, the ability of cells to activate and maintain cytoprotective autophagy via TFEB may be impaired, while cell proliferation and malignancy is promoted. Altogether, our study demonstrates that XPO1 function is conserved across phyla and may serve as an interesting therapeutic target to stimulate autophagy and prevent age-related diseases.
